# CCR5Δ32 in Brazil: Impacts of a European Genetic Variant on a Highly Admixed Population

**DOI:** 10.3389/fimmu.2021.758358

**Published:** 2021-12-10

**Authors:** Bruna Kulmann-Leal, Joel Henrique Ellwanger, José Artur Bogo Chies

**Affiliations:** Laboratório de Imunobiologia e Imunogenética, Programa de Pós-Graduação em Genética e Biologia Molecular (PPGBM), Departamento de Genética, Universidade Federal do Rio Grande do Sul (UFRGS), Porto Alegre, Brazil

**Keywords:** CCR5, CCR5delta32, Brazil, cancer, inflammation, infectious disease, pathogen, population genetics

## Abstract

The genetic background of Brazilians encompasses Amerindian, African, and European components as a result of the colonization of an already Amerindian inhabited region by Europeans, associated to a massive influx of Africans. Other migratory flows introduced into the Brazilian population genetic components from Asia and the Middle East. Currently, Brazil has a highly admixed population and, therefore, the study of genetic factors in the context of health or disease in Brazil is a challenging and remarkably interesting subject. This phenomenon is exemplified by the genetic variant CCR5Δ32, a 32 base-pair deletion in the *CCR5* gene. CCR5Δ32 originated in Europe, but the time of origin as well as the selective pressures that allowed the maintenance of this variant and the establishment of its current frequencies in the different human populations is still a field of debates. Due to its origin, the CCR5Δ32 allele frequency is high in European-derived populations (~10%) and low in Asian and African native human populations. In Brazil, the CCR5Δ32 allele frequency is intermediate (4-6%) and varies on the Brazilian States, depending on the migratory history of each region. CCR5 is a protein that regulates the activity of several immune cells, also acting as the main HIV-1 co-receptor. The CCR5 expression is influenced by CCR5Δ32 genotypes. No CCR5 expression is observed in CCR5Δ32 homozygous individuals. Thus, the CCR5Δ32 has particular effects on different diseases. At the population level, the effect that CCR5Δ32 has on European populations may be different than that observed in highly admixed populations. Besides less evident due to its low frequency in admixed groups, the effect of the CCR5Δ32 variant may be affected by other genetic traits. Understanding the effects of CCR5Δ32 on Brazilians is essential to predict the potential use of pharmacological CCR5 modulators in Brazil. Therefore, this study reviews the impacts of the CCR5Δ32 on the Brazilian population, considering infectious diseases, inflammatory conditions, and cancer. Finally, this article provides a general discussion concerning the impacts of a European-derived variant, the CCR5Δ32, on a highly admixed population.

## Introduction

### Genetic Aspects of the Brazilian Population

Until the year 1500 CE, Brazil was inhabited only by Native Americans belonging to different linguistic groups, distributed along the coast and hinterland of the country. This scenario changed dramatically after the arrival of the Portuguese explorers in the Brazilian territory that year, affecting many cultural and biological aspects of the native populations. The European colonization of Brazil and the associated influx of Africans had a strong influence on the genetic makeup of the Brazilian population. In Brazil, as well as in other countries colonized by the Europeans, the Native American population deeply declined after colonization (contracted around 90% in the Americas) (1–3). The remaining native population underwent a strong process of genetic miscegenation. However, the processes of population change continued throughout Brazilian history, even in more recent times. Over the past 200 years, Brazil has received a large influx of European immigrants from various countries, also described as the last migration pulse, which added another layer to the genetic makeup of the Brazilian population (1–4).

In general terms, the genetic background of current Brazilians has Amerindian, African, and European components in different proportions (2, 3, 5–7), depending on the Brazilian region under investigation (North, Northeast, Center-West, Southeast, or South). For example, the genetic makeup of Brazilians in the southern region of Brazil was strongly influenced by migratory flows from Europe in the 19th and 20th centuries; although in the Northeast of the country, the African genetic component is high (1, 2, 8). Of note, the European component is preponderant in different Brazilian regions when the Amerindian, African, and European components are compared, but even observing some regional peculiarities as those mentioned above, the genetic composition of the Brazilian population is rather uniform in its miscegenation in different regions of the country (1).

Throughout history, Brazil also received migrants from other countries beyond those from Europe and Africa, including countries from Asia and Middle East (7, 9). The intense migration within the national territory (10) allowed the exchange of genetic information between Brazilians from different regions, ethnic and genetic groups. As a result of the interactions of these different groups, the Brazilian population is currently highly miscegenated, a characteristic evident in the rich genetic and phenotypic diversity observed among the Brazilian population (2, 6, 11, 12). Considering the scenario mentioned above, the Brazilian population can be considered genetically heterogeneous and admixed, in addition to being relatively uniform throughout the country (1). Interestingly, admixed Brazilian populations are probable “reservoirs” of the diverse Native American genetic component (3), currently the least prevalent genetic component in the population (1, 8).

Y-chromosome haplogroup analysis corroborates the high genetic miscegenation observed in the Brazilian population. Abe-Sandes et al. (13) investigated the frequency of different haplogroups in Brazilian individuals from different ethnicities. A significant frequency of typical European haplotypes in Afro-Brazilians was found, for example, in the Quilombola community of São Gonçalo, Bahia state, northeastern Brazil. Abe-Sandes et al. (13) also found the E-SRY4064 haplotype, usually observed in populations from Sub-Saharian Africa and almost absent in populations from Europe and Asia, in white Brazilians, in a notable frequency (13). Marrero et al. (14) also reported evidence of admixture in Native American populations, showing the presence of non-Amerindian haplotypes in Kaingang and Guarani peoples (14). Finally, numerous studies analyzing Y-chromosome haplogroups reinforce the miscegenation addressed in this article, pointing to European, Amerindian, African and Asian haplogroups in different ethnicities and population groups from different Brazilian regions (13–28).

In the same direction, evaluation of mitochondrial DNA in different populations of Brazil showed the presence of diverse haplogroups characteristic of African, European, Native American and Asian populations, again evidencing the high level of miscegenation in the Brazilian population (14, 29–37). Of note, Cardena et al. (38) assessed a population from São Paulo, southeastern state of Brazil, specifically evaluating mtDNA haplogroups and comparing such data with self-declared ethnicity. Interestingly, a significant parcel of the individuals classified as whites showed a high percentage of African mtDNA (37.6%), with less participation of Amerindian (31.6%) and European (30.8%) origins. When analyzing other genomic loci of the same individuals, a higher European contribution was noticed (63.3%), evidencing a considerable African participation of maternal origin in individuals simultaneously presenting high non mtDNA European ancestry (38, 39).

### Pivotal Information Regarding the CCR5Δ32 Variant

The CCR5Δ32 polymorphism (reference SNP ID number: rs333) is a genetic variant that originated in the European population (40), and therefore can be used as an ancestry-informative marker in studies involving population genetics and genome ancestry (41, 42). This variant represents a 32-base pair deletion in the *CCR5* gene (chromosome 3; 3p.21.31), a fundamental component of the immune system responsible for encoding the CCR5 protein, which acts mainly in the regulation of inflammatory cell migration. It is unclear what selective pressures (considering positive selection) were responsible for fixing CCR5Δ32 in the human genome. Smallpox, bubonic plague, and other infectious diseases have already been suggested, but there is no consensus on this aspect (40). Neutral evolution is also a possibility (43). What is somehow certain is that the variant probably originated in the European population at 700-5,000 years ago (43, 44), potentially even earlier than 5,000 years (45, 46), and later spread heterogeneously across the world.

The CCR5Δ32 allele presents a higher frequency in northern Europe (greater than 15% in Norway, Latvia, and Estonia), being less frequent in countries located in the south of the European continent. For example, the frequency of the CCR5Δ32 allele is 8.1% in Spain, 6.9% in Portugal, 6.2% in Italy, and 5.1% in Greece. The allele frequency is very low or even absent in most Asian and African countries: for example, 0.4% in China, 2.2% in Korea, 0.7% in Cameroon, 0.26% in Eritrea, and 2.9% in Egypt (47). A recent study reports the absence of the CCR5Δ32 allele in the Nepalese population (48). Similarly, CCR5Δ32 is rare in Native American groups, showing an overall CCR5Δ32 allele frequency of 0.2%, mostly probably due to miscegenation (42). In the contemporary Brazilian population, the overall frequency of the CCR5Δ32 allele usually ranges from 4 to 6% but showing significant variations between different Brazilian regions and ethnic groups (42, 49), as will be discussed in the next sections of this article.

The main function of the CCR5 is coordinating leukocyte migration during inflammatory reactions through interaction with different chemokines, especially CCL3, CCL4, and CCL5 (40). Of note, these chemokines were historically called “MIP-1α”, “MIP-1β” and “RANTES”, respectively, but that denomination has fallen into disuse (50, 51). The CCR5 protein is expressed on the cell surface and has seven transmembrane domains connected by three extracellular loops and three intracellular loops. Leukocytes are the main cells that express the CCR5 (40), although the protein is also detected in other cell types, such as human embryonic neurons (52), adipocytes (53), and several types of cancer cells and tissues (54–58), indicating that CCR5 performs immune functions that go beyond coordinating the migration of inflammatory cells.

Carriers of the wild-type *CCR5* gene have CCR5 expression constitutively, with some variation between individuals. CCR5Δ32 causes important phenotypic effects, affecting the interaction of the CCR5 with chemokines. Due to the induction of a change in the *CCR5* gene reading frame, the CCR5Δ32 produces a truncated protein that is not expressed on the cell surface, presenting a gene-dosage effect. In brief, the presence of the CCR5Δ32 allele in heterozygous causes a reduction in the expression of CCR5 at the membrane. The presence of the CCR5Δ32 allele in homozygosis culminate in virtually no expression of CCR5 molecules on the cell surface (59–63). The CCR5Δ32-derived molecules are not phosphorylated and remain retained in the endoplasmic reticulum (64). Interestingly, it was suggested that in addition to the gene-dosage effect associated to CCR5Δ32, the CCR5Δ32-derived truncated protein could promote the sequestration of the CCR5 and CXCR4 proteins, both HIV-1 co-receptors, from the cell surface (65, 66).

These changes in the expression of CCR5 associated to CCR5Δ32 culminate in a disrupted CCR5-mediated immune response, which can be beneficial in some situations or harmful in others (67) since the ‘chemokine system’ is not completely redundant. The absence of CCR5 can impact the cell signaling coordinated by CCL3, CCL4 and CCL5, thus perturbing the proper CCR5-mediated immune responses (68). Disruptions in the chemokine system can significantly alter the susceptibility and progression of different diseases. For instance, COVID-19 severe cases are associated with uncontrolled receptor-ligand interactions and consequent inflammatory dysregulation, which characterizes the cytokine storm frequently observed in such severe disease cases (69, 70). Recently, CCR5Δ32 deletion was identified as a protective factor in Czech First-Wave COVID-19 subjects (71). Different *CCR5*-editing techniques are currently available and can be used to test *in vitro* the impacts of the CCR5 absence in different conditions, simulating the consequences of CCR5Δ32 on the immune system and disease conditions (72, 73). However, it is essential to emphasize that the *CCR5*-editing in human embryos raises many ethical concerns and may have deleterious consequences (67, 74).

**Figure 1:**
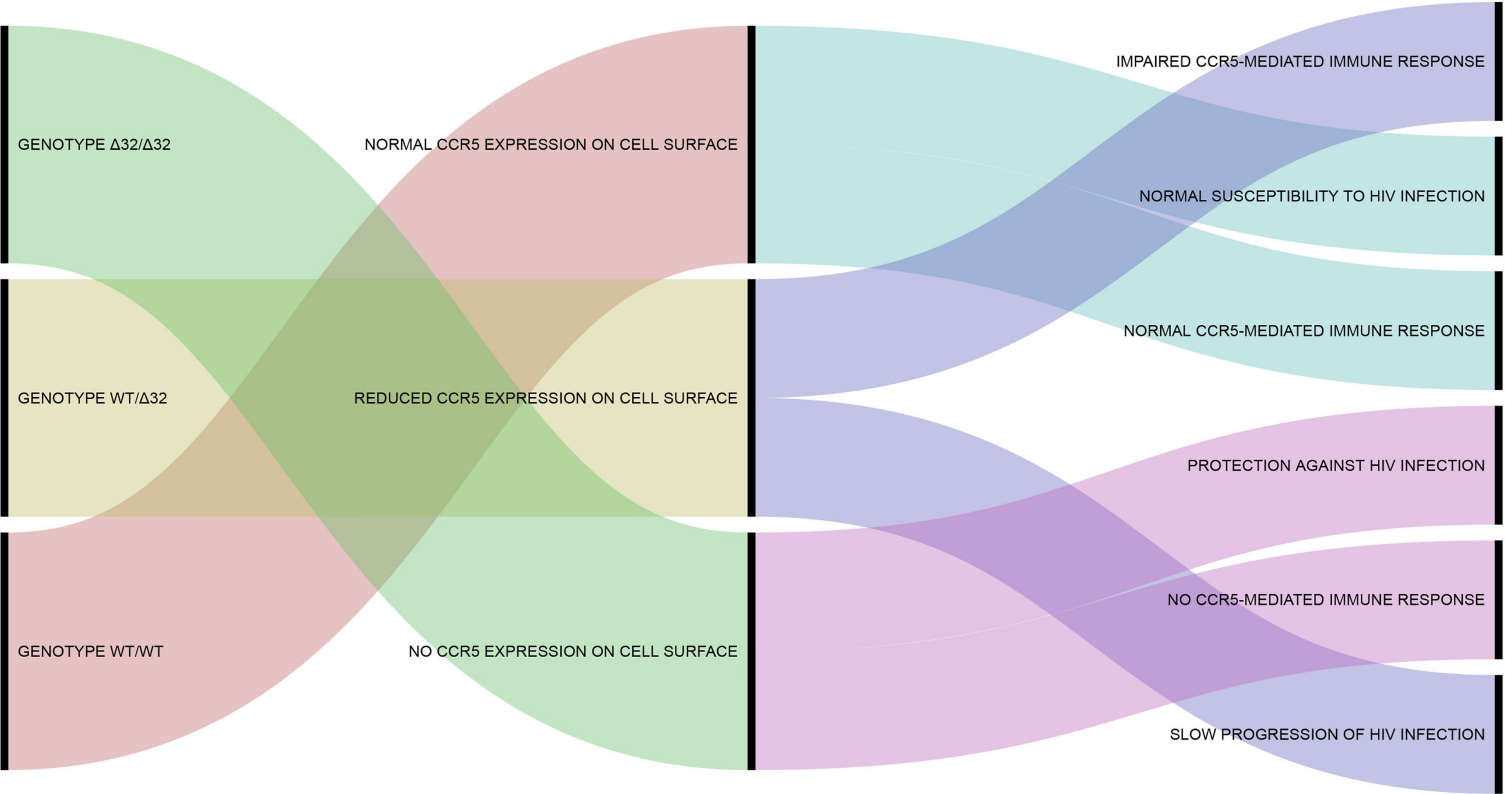
Alluvial diagram representing the classic outcomes associated with the CCR5Δ32. The CCR5Δ32 genotypes are shown in the left part of the diagram. The phenotypic effects of each genotype are shown in the center. The more classical consequences associated with each phenotype are shown in the right part of the diagram. Additional information concerning the phenotypic effects of the CCR5Δ32 on human cells and immune system can be found in previous studies of our group (40, 68, 81). This figure was created using RAWGraphs (https://rawgraphs.io/) (82).

Looking at the desirable effects, CCR5Δ32 protects against HIV infection, since the homozygous state of the variant impairs the proper expression of CCR5, preventing the interaction of CCR5 (the main HIV co-receptor) with the virus on the cell surface, thus avoiding infection of the host (75, 76). As mentioned above, CCR5Δ32-derived molecules (CCR5 truncated proteins) can also have an important protective effect against HIV by sequestrating CCR5 and CXCR4 from cell surface (65, 66). The discovery of this effect was truly relevant because it gives support to the use of CCR5 blockers for the clinical control of HIV infection. The best example of this case is maraviroc, a noncompetitive CCR5 antagonist that prevents the proper interaction between the HIV envelope glycoprotein and the CCR5. Currently, other CCR5 blockers (e.g., cenicriviroc, leronlimab) are being tested to treat HIV infection and other inflammatory conditions, and maraviroc emerges as a potential drug to treat other diseases involving CCR5, especially some types of cancer (77). In Brazil, CCR5 blockers represent a good choice for HIV treatment, since most of the circulating viral strains show CCR5 tropism (78–80). Based on the scenario presented above, **Figure 1** shown an alluvial diagram representing the classic outcomes associated with the CCR5Δ32, including “desirable” and “undesirable” effects.

Another major achievement involving CCR5Δ32, and HIV infection was the sustained remission of the infection in the ‘Berlin Patient’, reported in 2009 (83) and confirmed in 2011 (84), and in the ‘London Patient’, reported in 2019 (85) and confirmed in 2020 (86). Both individuals were HIV positive and developed hematological malignant diseases (acute myeloid leukemia and Hodgkin’s lymphoma, respectively), requiring allogeneic hematopoietic stem-cell transplantations. After receiving cell transplantations from CCR5Δ32 homozygous donors, both showed sustained remission of HIV infection. Other cases like Berlin and London patients are being followed up, such as the ‘Düsseldorf patient’ (87). The success of this strategy, although involving few cases, shows that sustained remission of HIV is possible to be achieved and subsequently maintained free of antiretroviral therapy. The Berlin patient, Timothy Ray Brown, passed away on September 29, 2020, due to the recurrence of acute myeloid leukemia, not HIV infection (88, 89). In addition to having collaborated enormously to advance research involving HIV, T. R. Brown created the Timothy Ray Brown Foundation and contributed significantly to the field of HIV/AIDS research, with a big and admirable impact on global society as an HIV activist (89–91).

Currently, it is known that the influence of CCR5 and CCR5Δ32 goes beyond protection against HIV infection and is much broader than previously believed, influencing the susceptibility and outcome of different conditions, such as other different viral, bacterial, and parasitic diseases (40, 92), as well as non-infectious inflammatory conditions (93–96). This occurs because the lack of CCR5 expression, in humans naturally due to CCR5Δ32, interferes with multiple aspects of inflammatory responses, including expression of immune system genes, levels of inflammatory markers, and activity of immune cells (97–103). On the other hand, now looking at the undesirable aspects of CCR5Δ32, this genetic variant increases the risk of serious complications caused by the West Nile virus and Tick-borne encephalitis virus (104–109).

Although Brazilians form a population of more than 210 million individuals, genetic studies in this population are still limited, with most genetic studies focusing on populations with European ancestry (6, 9). The Brazilian population can serve as a study case to understand the impact of genetic admixture on the frequency of genetic variants, such as CCR5Δ32, and its impacts on different conditions and pharmacogenomics (7). Understanding the extent to which the CCR5Δ32 variant influences the health of different populations is critical since it indicates which individuals and ethnic groups are more likely to benefit from therapies focused on modulating CCR5 in the context of cancer, infections, and inflammatory diseases. Focusing on HIV, knowing the frequency of CCR5Δ32 in different human populations is the initial step to guide potential new attempts at sustained remission of HIV infection through stem cell transplantation with CCR5Δ32 homozygous genotype. Moreover, it is also essential to understand how CCR5Δ32 impacts the health of the Brazilian population.

Considering that (I) the frequency of CCR5Δ32 is quite varied among Brazilians from different country’s regions and that (II) the role of CCR5Δ32 in various pathological conditions is an emerging topic with several knowledge gaps, the primary objective of this article is to review the effects of the genetic variant CCR5Δ32 on the Brazilian population, considering several diseases and clinical conditions. The secondary objective of this article is to discuss the impacts of a European-derived variant, the CCR5Δ32, on a highly mixed population.

## Methods

For the initial selection of articles, the terms “CCR5”, “CCR5 delta 32”, “CCR5Δ32” and “rs333”, used in combination with “Brazil” or “Brazilian”, were searched on PubMed (https://pubmed.ncbi.nlm.nih.gov/). Subsequently, the same search strategy was used on Scientific Electronic Library Online - SciELO (https://scielo.org/). The articles were initially selected based on the title and abstract. Only articles addressing CCR5Δ32 in Brazilian populations were included in this review. Articles published in English and Portuguese were considered in the evaluation, without restriction concerning the date of publication. On some specific occasions, the reference list of selected articles was also used as an additional source of published works involving CCR5Δ32 in the Brazilian population. Additional unstructured searches were performed on PubMed to select the articles cited in the introduction section and additional points of the review.

## CCR5Δ32 Frequency in Brazil

**Figure 2:**
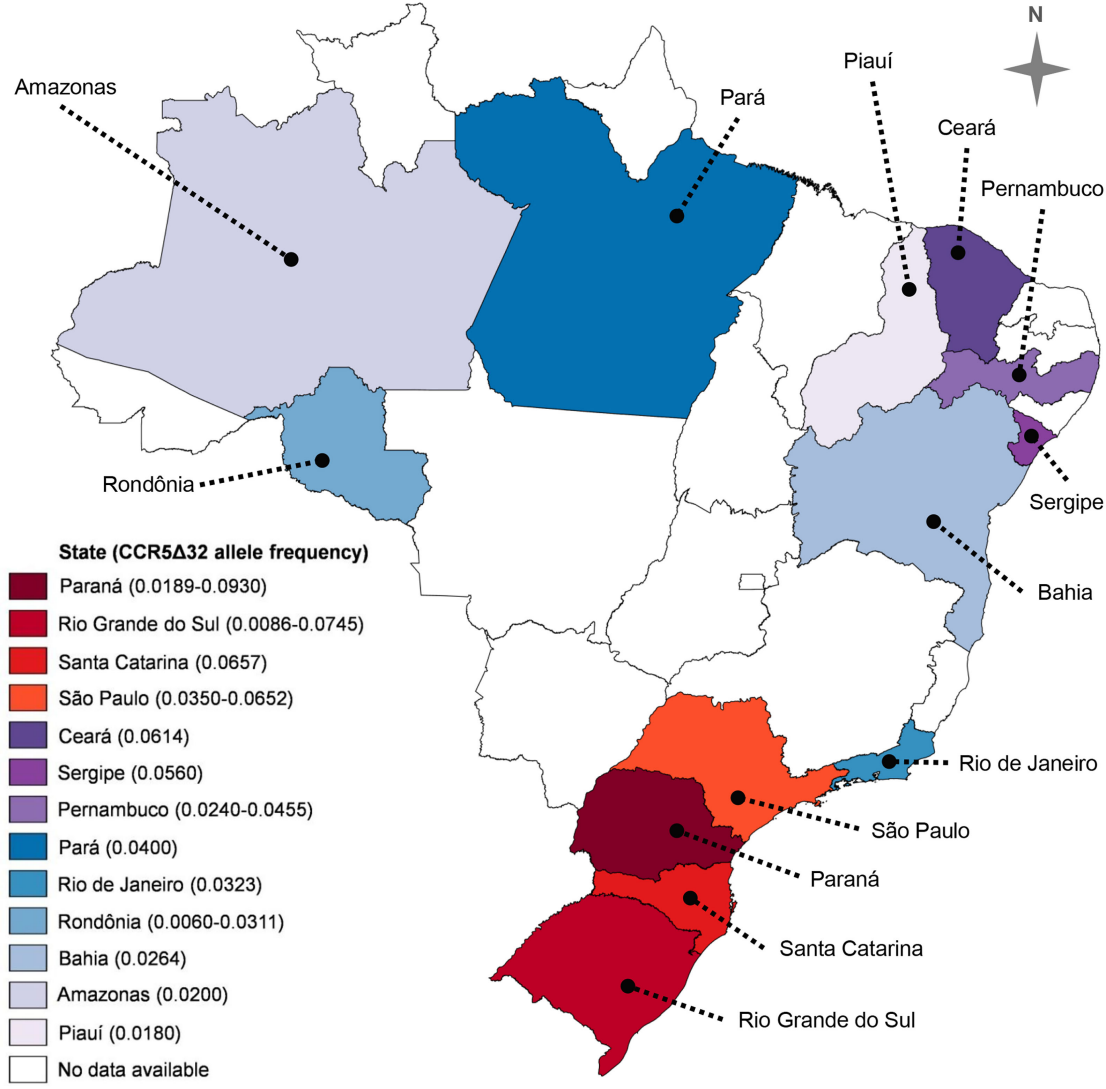
CCR5Δ32 allele frequency in thirteen Brazilian states. Two values in parentheses represent the lowest and the highest frequency observed in a given state. Data from Silva-Carvalho et al. (49), Hüneimeier et al. (110) (Mura population; Amazonas State), Carvalho et al. (41) (Mocambo community; Sergipe State), and Ferreira-Fernandes et al. (111) (Piauí State). The map was created with the help of MapChart (https://mapchart.net/), licensed under a Creative Commons Attribution-ShareAlike 4.0 International License.

A study published in 2016 by Silva-Carvalho and collaborators (49) presented a very complete meta-analysis regarding the CCR5Δ32 frequency in Brazil. In addition to original data from those authors, the meta-analysis included 29 articles reporting the CCR5Δ32 frequency in Brazil, encompassing populations from ten Brazilian States. The study found an overall allelic frequency of 4% in the country (49). The frequencies of the CCR5Δ32 allele in the Brazilian States, including data compiled by Silva-Carvalho et al. (49), are summarized in **Figure 2**. Henceforward, we expand the information concerning the CCR5Δ32 frequency in Brazil, highlighting studies not included in the meta-analysis by Silva-Carvalho et al. (49), and including data obtained from studies with indigenous populations and quilombola communities, as discussed below.

Leboute et al. (112) reported the absence of the CCR5Δ32 allele in a sample of 300 Amerindians from four indigenous populations of the Brazilian Amazon region, namely: Tikuna (n = 191), Baniwa (n = 46), Kashinawa (n = 29), and Kanamari (n = 34). Based on such data, we can argue that, at least until the date of publication of that work, the studied Amazonian tribes probably did not have a significant degree of miscegenation at a level sufficient for the introduction of the CCR5Δ32 allele into those indigenous groups. Alternatively, the allele could already be circulating in the groups, but it may not have been detected due to the small sample size (112).

Carvalhaes et al. (113) also described the frequency of the CCR5Δ32 allele in different ethnic groups of the Brazilian Amazon region, specifically from Pará State. The sample groups investigated were composed of 394 individuals from Belém (capital of Pará), 67 Afro-Brazilian individuals, 89 Amerindian individuals, and 111 Japanese immigrants. The CCR5Δ32 allele was not observed in Amerindian individuals and Japanese immigrants. In the sample of Afro-Brazilian individuals, only one individual carrying the allele in heterozygous was found, with the allele frequency, in this case, being 0.75%. In the sample of random individuals from Belém, one homozygous individual for the gene deletion and 22 heterozygous individuals were found, resulting in a CCR5Δ32 allele frequency of 3.04% (113).

Hünemeier et al. (110) evaluated the frequency of the CCR5Δ32 allele in Native American populations in Brazil and Paraguay: five Amazonian groups (Tiriyo, Mura, Cinta Larga, Gavião, and Zoró); a group from the Paraguayan Gran Chaco (Lengua); one from the Paraguayan forest (Aché); and one from southern Brazil (Kaingang). The CCR5Δ32 allele was found only in two groups: Mura (2%) and Kaingang (3%). The presence of the CCR5Δ32 allele in the samples of these two groups may be due to gene flow, which is explained by previous data showing that both populations have a degree of miscegenation. Thus, the CCR5Δ32 allele may have been introduced in American-native populations due to European miscegenation (110).

Vargas et al. (42) investigated the distribution of the CCR5Δ32 allele in individuals from Alegrete, a city in the western region of Rio Grande do Sul State. The population of Alegrete is highly admixed, with the genetic participation of Spanish, Portuguese, African, and Amerindian peoples. In the study, 103 healthy and unrelated individuals were analyzed, being divided into ‘white’ (n=59), ‘brown’ (n=31), and ‘black’ (n=13). No CCR5Δ32 homozygous individuals were found, and the frequency of heterozygotes was 14% in whites, 13% in browns, and 8% in blacks. Allele frequencies were 6.8%, 6.4%, and 3.8%, respectively (42). In Brazil, the classification of ethnicity performed by the government agency *Instituto Brasileiro de Geografia e Estatística* (Brazilian Institute of Geography and Statistics) is based on skin color, and for this reason many Brazilian studies classify individuals using this criterion. Alternatively, ‘white’ individuals can be classified as Caucasians, and ‘brown’ and ‘black’ can be classified as non-Caucasians.

Ferreira-Fernandes et al. (111) analyzed the CCR5Δ32 frequency in a sample of the population of the Piauí State. The sample consisted of 223 elderly individuals from the Network of Research on Frailty in Elderly Brazilians. The CCR5Δ32 allele was found only in heterozygous in the sample, with an allele frequency of 1.8%. In order to have a more robust investigation, the sample was also stratified according to sex and age (dividing the groups into individuals below or above 73 years old), but the frequencies were not statistically different between groups, ranging from 1.5% to 2.3%. The general CCR5Δ32 frequency observed is in accordance with other data presented by groups also from northeastern Brazil (111).

Carvalho et al. (41) evaluated the CCR5Δ32 frequency in three quilombola communities in the states of Sergipe (Mocambo community) and Bahia (Rio das Rãs and São Gonçalo communities). The groups were founded about 150 years ago by individuals from Sub-Saharan Africa and/or their descendants. The study evaluated individuals born in quilombola communities and recent immigrants, with a total of 100 inhabitants from Rio das Rãs, 71 from Mocambo, and 53 from São Gonçalo. In these communities, 28 were recent immigrants from Rio das Rãs, 18 from Mocambo, and 15 from São Gonçalo. Thus, the total sample size was 224 individuals: 163 born in the quilombos and 61 recent immigrants. In most cases, the oldest person in each family was chosen to participate in the study. The CCR5Δ32 allele was found in the three communities evaluated, but only in heterozygosis, with allele frequencies of 5.6% in Mocambo, 1% in Rio das Rãs, and 0.9% in São Gonçalo. According to the authors, the differences in allele frequencies can be due to several factors, including different proportions of parental populations in the founder’s individuals, a founder-effect, and different patterns of inter-ethnic contact (41).

Finally, we summarized in **Figure 2** the frequencies of CCR5Δ32 allele in thirteen Brazilian States, according to data of ten states compiled by Silva-Carvalho et al. (49), and the frequencies observed by Hüneimeier et al. (110) in the Mura population (Amazonas State), by Carvalho et al. (41) in individuals from Mocambo community (Sergipe State), and Ferreira-Fernandes et al. (111) in individuals from Piauí. To the best of our knowledge, there are no data available in the literature on CCR5Δ32 in the other Brazilian States.

## CCR5Δ32 in Infectious Diseases

CCR5 plays a critical role in the regulation of the immune response against infectious agents, controlling the traffic of immune cells [e.g., Natural Killer (NK) and T-regulatory (Treg) cells] towards inflammation sites. For instance, a recent study with mice showed that CCR5 has a pivotal role in the recruitment of NK cells to the kidney allowing an adequate neutrophil activity during systemic *Candida albicans* infection, acting as a fundamental molecule for a proper immune response. The absence of CCR5 expression resulted in uncontrolled inflammation and increased renal damage in face of *C. albicans* infection (114). Also, Treg cells play a fundamental role in resolving inflammatory conditions, providing an immunosuppressive activity. During infection by different pathogens (e.g., *Schistosoma* spp.), the poor recruitment of Treg cells to the inflammation sites due to CCR5 absence causes uncontrolled inflammation and related tissue damage (40, 115). On the other hand, during Rocio virus infection, the CCR5 absence was associated with reduced brain inflammation and better prognosis in animals (116). Taking together, imbalances in the CCR5-mediated immune responses due to CCR5Δ32 can cause both reduced and exacerbated inflammation, depending on the type of pathogen responsible for the infection (e.g., fungus, bacteria, virus), the infection site, or the immune cell type affected by the lack or reduction of CCR5 expression (40). In this context, studies addressing CCR5Δ32 and viruses in the Brazilian population will be discussed here, including HIV, Human T-lymphotropic virus (HTLV), Dengue, Influenza A, Hepatitis C virus (HCV), Hepatitis B virus (HBV), and Human papillomavirus (HPV).

As explained in the introduction section, CCR5Δ32 exerts its protective effect against HIV infection through two mechanisms: reduced expression of the *CCR5* gene (gene-dosage effect; probably the most important mechanism) (60, 63) and sequestration of CCR5 and CXCR4 from the cell surface (65, 66). Many studies that evaluated CCR5Δ32 in the Brazilian population corroborated the protective effect of the variant on susceptibility or clinical aspects of HIV infection (e.g., 117–120), although other studies have not evidenced these effects, in some cases probably due to the small sample size (e.g., 121, 122). The main results of the studies involving CCR5Δ32 and HIV infection in Brazil are detailed in **Table 1**.

**Table 1:** Impacts of the CCR5Δ32 on HIV infection.

Population	Sample	Main findings	Reference
Brazilian HIV+ individuals	177 ARV-naive individuals	Heterozygous individuals for CCR5Δ32 have a better response to ARV treatment than wild-type homozygotes	Accetturi et al. (117)
Brazilian individuals from different regions	1162 individuals (133 with HIV+ status)	CCR5Δ32 heterozygous cells (PBMCs) showed partial resistance to R5-HIV-1 *in vitro*; No significant differences in CD4+ T-cell counts between HIV+ individuals heterozygous and wild-type homozygous for CCR5Δ32; HIV load in heterozygous individuals are significantly lower than in wild-type individuals	Grimaldi et al. (123)
Individuals from São Paulo State, Brazil	129 HIV+ individuals and 26 blood donors	CCR5Δ32 heterozygous genotype was associated with reduces RANTES/CCL5 levels	Mikawa et al. (124)
Individuals from São Paulo State, Brazil	183 HIV+ individuals and 115 controls	The frequency of the CCR5Δ32 heterozygous genotype was lower in HIV+ individuals (11.5%) than in controls (13.0%)	Munerato et al. (125)
Individuals from Pará, Brazil	110 HIV+ and 139 uninfected individuals	Similar frequencies of the CCR5Δ32 allele were observed in the two groups: 2.7% in HIV+ individuals and 2.2% in the controls	Carvalhaes et al. (121)
Children from Pernambuco State, Brazil	106 HIV+ and 70 uninfected children exposed to infection risk and 104 controls	No significant influence of the CCR5Δ32 in the risk of HIV vertical transmission	Souza et al. (126)
HIV+ children from São Paulo State, Brazil	51 HIV+ children divided into rapid, moderate and slow progressors	No influence of the CCR5Δ32 in disease progression (limited sample size)	Angelis et al. (127)
Individuals from southern Brazil	134 blood donors; 145 HIV-exposed seronegative individuals; 152 HIV+ asymptomatic individuals; 478 HIV+ individuals with AIDS	CCR5Δ32 homozygous genotype was significantly associated with reduced risk of HIV infection	Vissoci Reiche et al. (118)
Individuals from São Paulo State, Brazil	200 HIV+ (155 on pre and post-ART) and 82 uninfected individuals	CCR5Δ32 heterozygous genotype was associated with better CD4+ T cell recovery after ART initiation	Rigato et al. (119)
Injecting drug users from Rio de Janeiro State, Brazil	48 HIV+ and 558 uninfected injecting drug users	No significant impact of the CCR5Δ32 on susceptibility or protection to HIV infection	Teixeira et al. (128)
Individuals from Bahia State, Brazil	506 HIV+ individuals (155 divided into rapid, typical and slow progressors)	CCR5Δ32 allele was more frequent in typical than in rapid progressors (without statistical significance)	Abe-Sandes et al. (122)
HIV+ individuals from Rio Grande do Sul State, Brazil	249 HIV+ individuals	CCR5Δ32 heterozygous genotype was associated with reduced risk of CD4+ T cell depletion (univariate analysis) and with increased risk of death after AIDS diagnosis (multivariate analysis; potentially due to the emergence of CXCR4-tropic HIV strains); CCR5Δ32 was a protective factor on disease progression in survival curve analysis	Vieira et al. (129)
Serodiscordant couples from Santa Catarina State, Brazil	9 HIV-exposed seronegative individuals; 9 ART-treated HIV+ individuals; 12 healthy controls	The CCR5Δ32 heterozygous genotype was observed in two HIV-exposed seronegative individuals, two ART-treated HIV+ individuals, and one control; In one serodiscordant couple, both individuals had CCR5Δ32 heterozygous genotype and the CXCR4 viral tropism was observed in the infected individual	Santos et al. (130)
Individuals from Roraima State, Brazil	117 HIV+ individuals	CCR5Δ32 heterozygous genotype was found in 11 individuals (9.4%); CCR5Δ32 allele frequency estimated at 4.6%	Corado et al. (131)
Individuals from Pernambuco State, Brazil	213 HIV+ and 234 uninfected individuals	CCR5Δ32 frequency was reduced in HIV+ individuals compared to controls; Stratification of data according to CCR5Δ32 genotypes did not modify the results of *TRIM5* polymorphisms observed in the study	Celerino da Silva et al. (132)
Individuals from São Paulo State, Brazil	66 HIV+ individuals with recent infection	CCR5Δ32 heterozygous genotype was detected in two individuals (one infected by R5-tropic HIV strain and other by CXCR4-tropic HIV strain); No significant association between CCR5Δ32 and tropism switch	Arif et al. (133)
Individuals from Paraná State, Brazil	35 individuals with HIV/HBV or HIV/HCV co-infection	CCR5Δ32 allele was not observed in the sample	Avanzi et al. (80)
Individuals from Pará State, Brazil	30 HIV+ individuals (divided into viremia controllers and non-controllers)	CCR5Δ32 heterozygous genotype was detected in one non-viremia controller	Gomes et al. (134)
Individuals from Paraná State, Brazil	81 perinatally infected HIV+ adolescents and young adults (61 genotyped for CCR5Δ32)	CCR5Δ32 heterozygous genotype was detected in one individual (1.6%); This patient was infected by an R5 HIV strain	Martin et al. (135)
Individuals from Pernambuco State, Brazil	266 HIV+ and 223 uninfected individuals	CCR5Δ32 frequency was reduced in HIV+ individuals compared to controls (without statistical difference); CCR5Δ32 along with other polymorphisms did not show statistically significant influence on plasma viral load	Celerino da Silva et al. (136)
Individuals from Rio Grande do Sul State, Brazil	294 uninfected individuals and 206 HIV+ individuals (divided into 40 rapid progressors and 166 non-rapid progressors)	Plasma viral load was lower among CCR5Δ32 heterozygous individuals as compared to wild-type homozygous individuals	Valverde-Villegas et al. (120)
Individuals from Pernambuco State, Brazil	248+ individuals divided into immunological recovery profiles during ART (222 of the 248 HIV+ individuals were genotyped for CCR5Δ32)	CCR5Δ32 heterozygous genotype was statistically associated with immunological recovery failure (result from logistic regression analysis)	Carvalho-Silva et al. (137)

ART: antiretroviral therapy.

Experimental evidence indicated that the course of HTLV (type 1 and 2) infection and HIV/HTLV co-infection may be affected by CCR5 expression patterns, which can be modulated by such viruses (138, 139). The CCR5 and its ligands can also influence the course of Dengue infection (140, 141). CCR5Δ32 was associated with an increased risk of fatal Influenza virus infection in Spanish individuals (142). However, CCR5Δ32 has a limited impact on these infections in the Brazilian population. Studying HTLV-1 infection, no statistically significant association was found between CCR5Δ32 and susceptibility or presence/absence of a symptomatic infection (143). Only one study was found regarding this evaluation in a non-Brazilian population. Hisada et al. (144) investigated the CCR5Δ32 frequency in Jamaican HTLV-1-infected individuals and healthy controls. However, the frequency found was too low to further conclusions. That said, no study found an association between the variant and HTLV-1 infection (144). Also, no statistically significant association was observed when the frequencies of CCR5Δ32 were compared between severe Dengue cases and controls (145). A similar study carried out in an Australian population also found no association between the CCR5Δ32 allele and DENV infection (146). The CCR5Δ32 was not associated with hospitalization in individuals infected by Influenza A virus (2009 pandemic H1N1 strain) (147). Subsequently, a study addressing the same virus also reported no significant effect of CCR5Δ32 on H1N1 infection severity (148). A study conducted in a Spanish population identified an association between the CCR5Δ32 allele and fatality due to Influenza A (H1N1) infection (142). Also, an association of the variant with disease severity was observed in a Canadian population (149). Therefore, further studies evaluating the role of this polymorphism in Influenza virus infection are needed.

HCV and HBV are associated with the development of hepatocarcinoma and other liver diseases (150). CCR5 could affect both susceptibility to these viruses and associated diseases due to its regulatory role in inflammatory reactions. Our group evaluated the influence of CCR5Δ32 on susceptibility to HCV infection and HCV/HIV co-infection. In the same study, we also accessed the potential impact of the CCR5Δ32 on HCV-related fibrosis, cirrhosis, and hepatocarcinoma. In total, 1352 individuals were included in the study. No statistically significant associations of CCR5Δ32 with the evaluated criteria were observed (151). Looking at data reported in other populations [see discussion in reference (151)], we highlight that the association between the CCR5Δ32 variant and HCV infection can show important biases in some populations, and other studies corroborate our results showing a lack of association between the variant and HCV infection. Importantly, our work had the largest sample evaluated in the context of HCV infection (151).

More recently, we evaluated the influence of CCR5Δ32 on susceptibility to HBV infection and HBV/HIV co-infection in a study involving 1113 individuals. We found no significant effect of CCR5Δ32 on susceptibility to HBV mono-infection. On the other hand, the CCR5Δ32 allele exerted a protective influence on HBV/HIV co-infection. Of note, this result was potentially due to the known protective effect of CCR5Δ32 on HIV infection (92). In a study in the Indian population, the heterozygous genotype (WT/Δ32) was associated with a higher susceptibility to HBV infection, whereas in a study in the Iranian population, the variant was a protective factor against the infection (152, 153). Other studies carried out in different populations reported a lack of association between HBV infection and the CCR5Δ32 variant (154–156), which is in agreement with the major finding observed in our previous study (92).

HPV is strongly associated with the development of cervical cancer (157) and it was suggested that CCR5 could play a role in the context of HPV infection and related diseases. Nevertheless, Mangieri et al. (158) observed no significant effect of CCR5Δ32 on susceptibility to the infection or cervical lesions (158). Also, the CCR5Δ32 was not associated with infection by a particular HPV genotype (159). In contrast, in a Swedish population, the homozygous genotype for the variant was associated with an increased risk of HPV infection (160). Given the limited amount of data and the contradictory results concerning the involvement of CCR5 in HPV infection, further evaluation concerning the potential role of the CCR5Δ32 variant in the context of HPV infection and related diseases in Brazilian and other populations are needed.

The influence of CCR5Δ32 on parasitic diseases was also investigated in the Brazilian population, including Chagas disease, leishmaniasis, and toxoplasmosis. CCR5 can have two opposite effects on Chagas disease, a disease caused by *Trypanosoma cruzi* infection. CCR5 mediates the control of acute infection, assuming a favorable role for the host. In opposition, the increased expression of CCR5 during Chagas disease is associated with exacerbated inflammation and related cardiac complications (161). Thus, the levels of CCR5 expression are critical in the outcome of Chagas disease. However, two other studies found no association between the CCR5Δ32 variant and cardiac or digestive manifestations on chronic Chagas disease (162, 163). In a Peruvian population, the frequency of the Δ32 allele was not high enough to allow an analysis of association with *T. cruzi* infection, and a study with individuals from Venezuela did not find an association of the variant with the presence of disease symptoms (164, 165). Therefore, the potential CCR5Δ32 allele role in Chagas disease is still under discussion.

Brajão de Oliveira et al. (166) and Ribas et al. (167) reported no statistically significant difference between Leishmania-infected individuals and controls concerning CCR5Δ32 frequencies (166, 167). In the study performed by Brajão de Oliveira et al. (166), the CCR5Δ32 allele carriers showed a less severe spectrum of clinical manifestations, but without statistical significance (166). Ribas et al. (167) observed a higher frequency of the CCR5Δ32 polymorphism among a subgroup of patients with recurrent lesion, but this specific result was based on an exceedingly small cohort (167). Also, a study performed in a Pakistani population showed no association between the CCR5Δ32 variant and cutaneous leishmaniasis (168).

The CCR5Δ32 wild-type genotype in association with AA or AG genotypes (from the *CCR5* rs1799987 polymorphism, an intron A/G SNP) was associated with increased risk of ocular toxoplasmosis, potentially due to the persistent CCR5-mediated inflammation in individuals with normal CCR5 expression (169). Also evaluating Brazilians, Vallochi et al. (170) found no association between the CCR5Δ32 and ocular toxoplasmosis (based on a brief description; detailed data not described by such authors) (170). No other studies evaluating the role of this variant in the context of ocular toxoplasmosis in non-Brazilian populations were found.

Based on the studies discussed above, apart from the protective effect of CCR5Δ32 on HIV infection, the impacts of CCR5Δ32 on viral and parasitic infections in Brazilian populations seem quite limited (details of each study are presented in **Table 1** and **Table 2**). However, considering the recognized role of CCR5 in the regulation of inflammation, it is possible that potential influences of CCR5Δ32 on non-HIV infections have not been detected due to the small number of studies carried out in Brazil on these topics, many of them involving a small sample size.

**Table 2:** Impacts of the CCR5Δ32 on infectious diseases.

Disease/Infection	Population (Brazilian state)	Sample	Main findings	Reference
HTLV-I infection	Individuals from Minas Gerais State, Brazil	229 blood donors (50 HTLV-I seronegative individuals; 179 HTLV-I-infected individuals)	No statistically significant association was observed concerning CCR5Δ32 and HTLV-I infection	Pereira et al. (143)
Cutaneous leishmaniasis (*Leishmania* infection)	Individuals from Paraná State, Brazil	100 individuals with cutaneous leishmaniasis and 100 healthy controls	No statistical significant difference regarding CCR5Δ32 frequency between the two groups	Brajão de Oliveira et al. (166)
Cutaneous leishmaniasis (*Leishmania* infection)	Individuals from Paraná State, Brazil	111 individuals with cutaneous leishmaniasis and 218 controls	No statistically significant difference of the CCR5Δ32 frequency was observed between cases and controls	Ribas et al. (167)
Dengue virus infection	Individuals from Rio de Janeiro State, Brazil	87 severe children cases of Dengue and 326 controls	No statistical significant difference regarding CCR5Δ32 frequency between the two groups	Xavier-Carvalho et al. (145)
Chagas disease (*Trypanosoma cruzi* infection)	Individuals from São Paulo State, Brazil	85 Chagas disease patients with normal left ventricular systolic function; 43 Chagas disease patients with mild to moderate left ventricular systolic dysfunction; 40 Chagas disease patients with severe left ventricular systolic dysfunction	No statistical significant association between CCR5Δ32 and Chagas disease-related left ventricular systolic dysfunction	Oliveira et al. (162)
Chagas disease (*Trypanosoma cruzi* infection)	Individuals from São Paulo State, Brazil	109 patients with digestive form of Chagas disease; 131 patients with cardiac form of Chagas disease; 172 controls	No statistical significant influence of the CCR5Δ32 on digestive or cardiac form of Chagas disease, including left ventricular systolic dysfunction	Oliveira et al. (163)
Influenza A infection (2009 pandemic H1N1)	Individuals from northern and northeastern regions of Brazil	174 non-hospitalized Influenza-infected individuals and 156 hospitalized Influenza-infected individuals	No statistical significant impact of the CCR5Δ32 on infection severity	Maestri et al. (147)
HPV infection	Individuals from Pernambuco State, Brazil	139 HPV-infected women with cervical lesions and 151 HPV-infected women without cervical lesions	No statistical significant influence of the CCR5Δ32 on HPV-related cervical lesions or infection by specific HPV genotype	Santos et al. (159)
HCV infection, HCV/HIV co-infection and HCV-related hepatic diseases	Individuals from Rio Grande do Sul State, Brazil	674 HCV-infected individuals (stratified between 124 individuals without hepatic manifestation, 268 individuals with fibrosis, 190 individuals with cirrhosis and 92 individuals with hepatocarcinoma); 104 HCV/HIV co-infected individuals; 300 HIV-infected individuals; 274 controls	No statistical significant influence of the CCR5Δ32 on susceptibility to HCV infection, HCV/HIV co-infection or HCV-related hepatic manifestations	Ellwanger et al. (151)
Ocular toxoplasmosis (*Toxoplasma gondii* infection)	Individuals from São Paulo State, Brazil	160 individuals with ocular toxoplasmosis; 160 individuals with non-ocular toxoplasmosis; 160 controls	In association with AA or AG genotypes (from *CCR5* 59029 A/G SNP - rs1799987), the CCR5Δ32 wild-type genotype was associated with increased risk of ocular toxoplasmosis (based on multivariate logistic regression analysis)	Faria Junior et al. (169)
HPV infection	Individuals from Paraná State, Brazil	164 HPV-infected women and 185 control women	No statistically significant influence of the CCR5Δ32 on susceptibility to HPV infection or cervical lesions associated with HPV infection	Mangieri et al. (158)
Influenza A infection (2009 pandemic H1N1)	Individuals from South, Southeast and Northeast Brazilian regions (nine states in total)	153 individuals with influenza like illness; 173 individuals with severe acute respiratory infection; 106 fatal influenza-infection cases	No significant effect of the CCR5Δ32 on severity of Influenza virus infection or Influenza-linked mortality	Matos et al. (148)
HBV infection and HBV/HIV co-infection	Individuals from Rio Grande do Sul State, Brazil	335 HBV-infected individuals; 144 HBV/HIV co-infected individuals; 300 HIV-infected individuals; 334 controls	No significant effect of the CCR5Δ32 on susceptibility to HBV mono-infection; CCR5Δ32 was a protective factor on HBV/HIV co-infection	Ellwanger et al. (92)

Finally, the impact of the CCR5Δ32 on fungal infections is unknown in Brazilian populations and quite sparse in other human populations, and therefore research in this field is needed. Of note, Brazil is affected by several endemic mycoses, such as Dermatophytosis, Paracoccidioidomycosis, Histoplasmosis, and Cryptococcosis, among others (171). Understanding whether and how the CCR5Δ32 influences the susceptibility or clinical progression of these diseases can provide insights into the potential use of CCR5-based therapies for these diseases.

## CCR5Δ32 in Inflammatory Conditions

Considering the critical role of CCR5 in the regulation of the inflammatory response, several authors have been investigating the effect of CCR5Δ32 on conditions that have their susceptibility or clinical course affected by different types (e.g., systemic, local) and intensity of inflammation. In this topic, we review the role of CCR5Δ32 on the following inflammatory diseases or inflammation-related clinical conditions: multiple sclerosis, systemic lupus erythematosus, preeclampsia, rheumatoid arthritis, juvenile idiopathic arthritis, periodontitis, osteomyelitis, transplant rejection, and sickle cell disease. Details of each study are described in **Table 3** and discussed below.

**Table 3:** Impacts of the CCR5Δ32 on inflammatory conditions.

Disease/Condition	Population (Brazilian State)	Sample		Main findings	Reference
		Cases	Controls		
Multiple sclerosis (MS)	Paraná State	124 MS patients	127 healthy individuals	There was no statistically significant difference regarding the CCR5Δ32 allele between patients and controls, and no association was also found regarding clinical course and *CCR5* variants; A decreased disease progression was observed in patients bearing the CCR5Δ32 allele, with carrier presenting lower Expanded Disability Status Scale (EDSS) values	Kaimen-Maciel et al. (172)
	São Paulo State and Rio Grande do Sul State	261 MS patients	435 healthy individuals	Considering only Euro-Brazilians, the CCR5Δ32 allele frequency was significantly higher in healthy individuals than in MS patients (*p*=0.013). Also, there was a higher frequency of Δ32 homozygous and heterozygous individuals in controls than in patients (*p*=0.033)	Troncoso et al. (173)
Juvenile idiopathic arthritis (JIA)	Rio Grande do Sul State	101 JIA patients and 203 rheumatoid arthritis patients	104 healthy individuals	The frequency of the CCR5Δ32 variant was significantly higher (*p*=0.028) in JIA patients (0.094) than in controls (0.038)	Scheibel et al. (174)
Osteomyelitis	Ceará State	39 bone trauma with osteomyelitis cases	114 bone trauma without osteomyelitis cases	The frequency of the CC5Δ32 variant did not vary significantly, but patients with type I or type II fractures that carried the allele did not develop the disease	Souza et al. (175)
Periodontitis	São Paulo State	197 chronic periodontitis cases and 91 aggressive periodontitis cases	218 healthy individuals and 193 chronic gingivitis cases	The frequency of the CCR5Δ32 variant was significantly higher in patients with chronic gingivitis (0.11) than in chronic (0.058) (*p*=0.01) or aggressive periodontitis (0.055) (*p*=0.03)	Cavalla et al. (176)
Preeclampsia	Rio Grande do Sul State and Rio de Janeiro State	155 preeclampsia pregnancies	144 healthy pregnancies	The frequency of the CCR5Δ32 variant was significantly higher (*p*=0.047) in healthy women (0.14) than in pre-eclamptic women (0.07)	Telini et al. (177)
	Minas Gerais State	156 preeclampsia pregnancies	213 healthy pregnancies	The frequency of the CCR5Δ32 variant was significantly higher (*p*=0.047) in healthy women (0.045) than in pre-eclamptic women (0.016)	Kaminski et al. (178)
Rheumatoid arthritis (RA)	Rio Grande do Sul State	92 RA patients	160 healthy individuals	The frequency of the CCR5Δ32 variant did not vary significantly between the groups	Kohem et al. (179)
	Pará State	186 RA patients	206 healthy individuals	The frequency of the CCR5Δ32 variant was significantly higher in healthy individuals (0.075) than in RA patients (0.040) (*p*=0.016)	Toson et al. (180)
	Rio Grande do Sul State	361 RA patients	233 healthy individuals	The frequency of the CCR5Δ32 variant was significantly higher in healthy individuals (0.034) than in RA patients (0.011) (*p*=0.022)
	Pernambuco State	104 AR patients	154 healthy individuals	The frequency of the CCR5Δ32 variant did not vary significantly between groups
	São Paulo State	89 AR patients	83 healthy individuals	The frequency of the CCR5Δ32 variant did not vary significantly between groups
Sickle cell disease (SCD)	Rio Grande do Sul State and Pernambuco State	79 SCD patients	112 healthy afro-Brazilian individuals and 102 healthy euro-Brazilian individuals	The comparison of the CCR5Δ32 frequency between afro-Brazilian healthy individuals (0.013) and SCD patients (0.051) was of borderline significance (*p*=0.05)	Chies and Hutz (181)
	Rio Grande do Sul State	73 SCD patients	58 healthy individuals	The frequency of the CCR5Δ32 variant did not vary significantly between groups	Vargas et al. (182)
	Pernambuco State	483 pediatric SCD patients and 312 adult SCD patients	247 healthy individuals	The frequency of the CCR5Δ32 variant did not vary significantly between the groups	Lopes et al. (183)
	Bahia State	20 SCD patients	–	The CCR5Δ32 variant was not found in any patient evaluated	Nascimento et al. (184)
Systemic lupus erythematosus (SLE)	Rio Grande do Sul State	280 euro-Brazilian SLE patients and 87 afro-Brazilian patients	235 euro-Brazilian healthy individuals and 200 afro-Brazilian healthy individuals	The frequency of the CCR5Δ32 variant was significantly higher in healthy euro-Brazilian controls (0.075) than in euro-Brazilian SLE patients (0.027) (*p*=0.002); Patients carrying the CCR5Δ32 variant were predisposed to the development of class IV nephritis (*p*=7E-6)	Schauren et al. (185)
	Paraná State	169 SLE female patients	132 female healthy controls	The frequency of the CCR5Δ32 variant was significantly higher in patients (0.068) than in healthy controls (0.019) (*p*=0.0047). Euro-Brazilian individuals carrying the allele had a higher predisposition to the development of SLE than in afro-Brazilian individuals carrying the same variant (*p*=0.0286). Patients with heterozygous genotype presented a lower age of SLE onset and higher levels of anti-dsDNA antibodies when compared to individuals homozygous for the wild type allele (*p*=0.0293 and *p*=0.0255, respectively).	Baltus et al. (96)
Transplant rejection	Paraná State	86 kidney transplant patients with rejection episodes	160 kidney transplant patients without rejection episodes	No statistically significant difference was found in the CCR5Δ32 frequency between the groups (8.3% for individuals with rejection episodes; 6.3% for transplant recipients without rejection)	Cilião et al. (186)

Multiple sclerosis is an autoimmune, chronic, and inflammatory disease showing heterogeneity in clinical findings. Chemokines and chemokine receptors are molecules involved in the pathogenesis of multiple sclerosis (172, 194), and the CCR5Δ32 can influence different aspects of this disease, as shown in studies with non-Brazilian individuals (195–197). A meta-analysis carried out in 2014 evaluated the role of this variant in multiple sclerosis in different populations, and concluded that the CCR5Δ32 is not associated with susceptibility to the development of multiple sclerosis in Europeans, calling attention to the need for further studies involving other populations (198). In Australian individuals, this variant also did not show a protective role to multiple sclerosis (199). However, other studies have shown an association of the Δ32 allele with treatment response, disease severity, and susceptibility to multiple sclerosis (196, 200–202). In Brazil, only two papers explored the possible impact of the CCR5Δ32 on multiple sclerosis. Based on magnetic resonance imaging, Kaimen-Maciel et al. (172) observed a decreased disease progression in patients bearing the CCR5Δ32 allele (172). Subsequently, Troncoso et al. (173) described a statistically significant higher CCR5Δ32 allele frequency in Euro-Brazilian controls (7.4%) compared to Euro-Brazilian patients (3.3%), suggesting a protective role of the variant on the development of multiple sclerosis. Besides, the frequency of the CCR5Δ32 was higher in Euro-Brazilian patients with progressive multiple sclerosis than Euro-Brazilian patients with relapse remitting multiple sclerosis (173). Both studies carried out in Brazil show that the CCR5Δ32 variant can influence both the susceptibility and the clinical outcome of multiple sclerosis.

Systemic lupus erythematosus is a chronic inflammatory autoimmune disease characterized by the large production of autoantibodies, triggering generalized tissue damage. This disease has different clinical manifestations and a complex genetic influence, and chemokines and their receptors, such as CCR5, are implicated in the pathogenesis of lupus (96, 185, 203, 204). The CCR5Δ32 variant has already been studied in this context, being previously associated to protection against lupus development and, albeit in a contradictory manner, this polymorphism was also associated to susceptibility to nephritis in lupus patients (203, 204). In Brazil, two studies evaluated the CCR5Δ32 variant in lupus.

Schauren et al. (185) investigated the role of the CCR5Δ32 in healthy patients and controls of Rio Grande do Sul State (185). A lower frequency of the CCR5Δ32 allele was found in Euro-Brazilian patients (2.7%) compared to Euro-Brazilian controls (7.5%), suggesting a protective role of the variant against the development of systemic lupus erythematosus. However, in the same study, patients with the CCR5Δ32 allele had a greater predisposition to the development of class IV nephritis than patients without the allele, which suggests a more severe clinical outcome associated with the genetic variant (185).

Baltus et al. (96) evaluated the frequencies of the CCR5Δ32 in patients and controls in the Paraná State, also southern Brazil. Unlike the first study, the frequency of the CCR5Δ32 allele was statistically higher in patients (6.8%) than in controls (1.9%), suggesting the variant as a risk factor for systemic lupus erythematosus. Also, by stratifying the sample according to ethnicity, the researchers identified that Euro-Brazilian individuals carrying the CCR5Δ32 were more likely to develop systemic lupus erythematosus than Afro-Brazilian patients carrying the variant. In another analysis of the study, CCR5Δ32 carriers had a lower age of systemic lupus erythematosus onset and higher levels of anti-dsDNA antibodies. Thus, the CCR5Δ32 allele was associated with increased susceptibility to the development of systemic lupus erythematosus and severity in clinical outcomes (96). Studies performed in different populations have found no association between the variant and the development of systemic lupus erythematosus (205–208). Such divergence involving the results mentioned above deserves attention and, therefore, more studies in other populations are required.

Preeclampsia is a hypertensive gestational complication and an important cause of maternal-fetal mortality in Brazil. Relevant clinical findings of the disease, such as edema and proteinuria after the 20th week of pregnancy, are intricate with an excessive inflammatory process and endothelial dysfunction. In preeclampsia, increased systemic production of pro-inflammatory chemokines was observed, highlighting the role of the chemokine-ligand system in this condition (177, 178, 209). Two studies evaluating the CCR5Δ32 variant in preeclampsia were carried out in Brazil, both published by our group, but evaluating samples from different Brazilian regions. Firstly, Telini et al. (177) evaluated the frequency of the CCR5Δ32 in Brazilian women who developed preeclampsia and women who did not develop this condition during their pregnancies. The group of healthy women had a higher frequency of the CCR5Δ32 allele (14%) when compared to the group of women who developed preeclampsia (7%). The analysis revealed a protective role of the variant on preeclampsia development (177). More recently, Kaminski et al. (178) also investigated the role of CCR5Δ32 in women who developed preeclampsia and in women with healthy pregnancies (178). In accordance with the results of Telini et al. (177), healthy pregnant women also showed an increased CCR5Δ32 allele frequency (4.5%) compared to the group of pregnant women with preeclampsia (1.6%). Thus, the study corroborated the protective role of the CCR5Δ32 variant on preeclampsia development, endorsing the hypothesis that a reduced inflammatory millieu may contribute to a lower risk of developing preeclampsia (177, 178). A study conducted in a Turkish population found similar results, strengthening the conclusion here presented (210).

Rheumatoid arthritis is a systemic autoimmune disease characterized by progressive damage to the joints caused by chronic inflammation in the synovial fluid. Given the intense migration of immune cells to the inflammation sites, the role of CCR5 in rheumatoid arthritis appears to be of great importance (179, 180). In Brazil, two studies investigating the role of the CCR5Δ32 variant in rheumatoid arthritis were published. Kohem et al. (179) evaluated the frequency of the allele in healthy patients and controls from the Rio Grande do Sul State, and no statistically significant difference was found between the groups. Of note, the sample group was relatively small, with 92 patients and 160 healthy controls (179). Toson et al. (180) performed a similar study but evaluating the frequency of the CCR5Δ32 variant in different Brazilian populations, considering four different regions (south, southeast, northeast, and north). Two of the four sample groups, from southern and northern regions, showed a statistically significant difference between rheumatoid arthritis patients and healthy controls (4% vs. 7.5%; 1.1% vs. 3.4%, respectively), being precisely the groups with the largest sample sizes. The difference concerning the northeast region sample was not statistically significant but followed a similar trend to the groups in southern and northern. Only the southeastern sample deviated from the trend, with the small sample size possibly being the reason for the lack of statistical association. In sum, the study suggests a protective role for the CCR5Δ32 variant against the development of rheumatoid arthritis (180). A meta-analysis carried out in 2012 concluded that the variant may play a role in protection to rheumatoid arthritis in European populations, corroborating the data found in Brazil (211).

Juvenile idiopathic arthritis is a chronic inflammatory condition characterized in the synovial joints of young people up to 16 years of age (174, 212). Scheibel et al. (174) investigated the potential association of the CCR5Δ32 variant with juvenile idiopathic arthritis subtypes in a sample from Porto Alegre, southern Brazil. A statistically significant difference was found between patients (9.4%) and healthy controls (3.8%), especially considering the group of patients of the systemic juvenile idiopathic arthritis subtype (25%). The researchers conclude that the CCR5Δ32 variant, although not a risk factor for the development of juvenile idiopathic arthritis, contributes to the progression and clinical status of patients (174). Interestingly, the meta-analysis previously mentioned (211) also explored the role of the Δ32 allele in juvenile idiopathic arthritis, and concluded that the variant was a protective factor for this condition as well (211). A further study comprising children from different populations found an association between the heterozygous genotype and mild disease course, but no influence on susceptibility to disease development (213). That said, these controversial results evidence the importance of novel studies investigating the CCR5Δ32 variant in juvenile idiopathic arthritis.

Periodontitis is an oral disease characterized by a chronic infection accompanied by inflammatory processes, causing irreversible and progressive destruction of dental support structures. The CCR5-mediated immune responses affect multiple aspects of periodontitis. For instance, not only CCR5 and its ligands are important in the context of disease protection, but also influence periodontal destruction and bone resorption (176, 214–217). Cavalla et al. (176) investigated the CCR5Δ32 variant and its possible influence on periodontitis development. The CCR5Δ32 allele was significantly more frequent in individuals classified in the group of chronic gingivitis (11.1%) than in individuals with chronic periodontal disease (5.8%) or aggressive periodontal disease (5.5%). This result suggests a protective role of the variant concerning periodontitis (176). Other studies carried out in Taiwan and Germany found no association between the variant and periodontitis (218, 219). Considering the conflicting results, it is interesting to carry out further studies in other populations to better understand the role of CCR5 in the development of periodontitis.

Osteomyelitis is an infectious-inflammatory condition that can occur after bone trauma often following *Staphylococcus aureus* infection (175, 220). Souza et al. (175) evaluated the CCR5Δ32 frequency in patients who were admitted to a hospital in Fortaleza, northeastern Brazil, with bone trauma. The patients were prospectively studied to assess a possible development of osteomyelitis. There was no statistically significant difference between individuals who developed and those who did not develop the disease, but all patients with closed fractures (type I or type II) and who carried the CCR5Δ32 variant did not developed the condition. The researchers conclude that the lack of statistical significance observed in their study was probably due to the low sample size (175). No other studies regarding the potential role of the CCR5Δ32 in osteomyelitis were found in the literature.

The immune response and inflammatory processes that occur after an organ transplant are critical in the process of tissue rejection. Genetic variants related to the immune system can therefore influence the response to transplantation (186, 221–223). Studies carried out in non-Brazilian populations observed no association between the CCR5Δ32 allele and kidney transplant rejection (224–228). A study in a multicentric sample from Europe showed a higher survival rate after kidney transplantation in individuals with the CCR5Δ32 homozygous genotype (222). In Brazil, Cilião et al. (186) evaluated the CCR5Δ32 frequency in transplanted individuals who had episodes of rejection comparing to individuals who did not have such episodes. A sample of 246 patients was collected in a referral hospital in Londrina, Paraná State. However, the frequency of the CCR5Δ32 variant did not vary significantly between the groups (186).

Sickle cell disease is an inherited disorder caused by a single nucleotide substitution in the beta-globin gene. This mutation originated in Africa and is, therefore, more common in African populations and Afro-descendants. Sickle cell disease can be understood as a chronic inflammatory condition, which may be the cause of associated secondary complications. In this sense, high levels of inflammation in sickle cell disease patients are related to disease morbidity (181–184). A study in a population from Egypt found no association between the variant and sickle cell disease (229). In Brazil, four studies investigated the influence of the CCR5Δ32 variant in sickle cell disease, all detailed below.

Chies and Hutz (181) assessed the potential role of the CCR5Δ32 in severe and recurrent infections that could contribute to differentiated survival of sickle cell anemia patients. The study involved individuals from different ethnic groups and the frequencies of the CCR5Δ32 allele found were 4.4% in Euro-Brazilian controls, 1.3% in Afro-Brazilian controls, and 5.1% in sickle cell anemia patients. When comparing these frequencies between the different groups, no statistically significant difference was found. However, it is important to note that, considering the same ethnic background of the groups of patients and Afro-Brazilian controls, a difference in the allele frequency was evidenced, being the CCR5Δ32 allele three times more present in the group of sickle cell anemia patients. Given the low frequency of the allele in the sample of Afro-Brazilian controls, a 3-fold increase in the group of patients is quite important. The researchers suggested that the CCR5Δ32 allele was more frequent in the group of patients for conferring some advantages concerning the clinical course of the disease (181). As mentioned previously, sickle cell anemia can be considered a chronic inflammatory disease (93), and patients with the CCR5Δ32 allele would benefit from developing inflammatory responses at low levels. According to this hypothesis, the CCR5Δ32 allele was associated with an improvement in the general health status of the patients (93, 181).

Subsequently, Vargas et al. (182) evaluated CCR5Δ32 in sickle cell anemia patients from Porto Alegre, Rio Grande do Sul State. No statistically significant difference was observed in the study but, interestingly, the CCR5Δ32 allele was present only in the group of patients with a severe clinical course (when the pain rate was considered). Such data may indicate a trend towards the development of a severe clinical course associated with the CCR5Δ32 allele in sickle cell anemia patients (182). Lopes et al. (183) compared the CCR5Δ32 frequencies of two groups of patients (pediatric and adult) and between sick adults and healthy controls from Pernambuco, northeastern Brazil. There were no statistically significant differences in any of the comparisons made in the study (183). Finally, Nascimento et al. (184) evaluated the CCR5Δ32 frequency in sickle cell anemia patients from Bahia State. However, the CCR5Δ32 allele was not found in the study (184).

## CCR5Δ32 in Cancer

Chemokines and chemokine receptors have fundamental participation in both antitumor response and pathogenesis of cancer. The migration of regulatory immune cells to tumor sites can create an immunosuppressor environment proper for cancer development. Also, cancer cells can subvert the anti-tumor action of chemokine-ligand interactions (187–191). Of note, CD4+ T cells are important modulators of the immune response, acting as drivers for the action of effector cells. Some CD4+ regulatory T cells express the CCR5 molecule, being this a key receptor of the cellular response against tumor development. The presence of the CCR5Δ32 variant can impair the action of CCR5+/CD4+ T cells, influencing the risk of cancer development. In brief, chemokine receptors can assume multiple roles in different tumoral processes, and more investigation is needed to unravel the connections between CCR5 and cancer (101, 192). Two meta-analyses published in 2014 evaluated the possible role of the Δ32 allele in cancer. Ying et al. (230) found no association of the variant with risk of tumorigenesis, while Lee et al. (205) found an association of the allele with susceptibility to cancer in Indians, specifically concerning breast cancer (205, 230). Further studies found associations of the CCR5Δ32 variant with improved metastasis-free survival in breast cancer patients and, contradictorily, also with an increased risk for developing breast cancer (231–233). In Brazil, the possible role of the CCR5Δ32 variant in cancer has been addressed (**Table 4**) and the available data will be presented below.

**Table 4:** Impacts of the CCR5Δ32 on cancer.

Cancer type	Population (Brazilian state)	Sample	Main findings	Reference
Acute lymphoblastic leukemia (ALL)	Paraná State	79 ALL patients and 80 healthy controls	No statistically significant differences regarding CCR5Δ32 between ALL patients and controls	Oliveira et al. (187)
Breast cancer (BC)	Paraná State	72 BC patients and 90 healthy women	The allelic frequency estimated in patients was of 3.47% and 7.78% in healthy women; However, no statistically significant difference was found between these groups	Aoki et al. (188)
Breast cancer (BC)	Paraná State	118 BC patients and 180 healthy women	No statistically significant differences between groups regarding susceptibility, clinical outcome, or treatment response.	Banin-Hirata et al. (189)
Breast cancer (BC)	Paraná State	94 samples from 47 BC patients (47 tumoral tissues and 47 adjacent tissues)	No impact of CCR5Δ32 on CCL5 levels considering tumoral or normal tissues	Derossi et al. (190)
Cervical intraepithelial neoplasia (CIN)	Pernambuco State	290 HPV+ women (151 without cervical lesions and 139 with cervical lesions, divided in 12 women with cervical cancer (CC), 40 women with CIN I and 87 with CIN II or III)	No statistically significant differences regarding CCR5Δ32 between CIN or CC patients and HPV+ women without lesions	Santos et al. (159)
Neuroblastoma (NB)	Paraná State	28 tissue samples from NB patients and 80 cancer-free children	CCR5Δ32 was more frequent in the group of NB patients than in healthy controls (*p*<0.05)	Vieira-Filho et al. (191)
Prostate cancer (PCa)	Paraná State	30 advanced PCa patients	Significant increase in CD3+ and CD4+ cells was observed in CCR5Δ32 non-carriers; The average CD4+/CD8+ cell ratio decreased in CCR5Δ32 non-carriers after treatment	Magnani et al. (192)
Prostate cancer (PCa)	Rio Grande do Sul State	119 healthy individuals, 136 PCa patients and 130 benign prostatic hyperplasia (BPH)	CCR5Δ32 allele was not statistically associated with risk of developing BPH or PCa or clinical outcomes of both conditions	Zambra et al. (193)

The action of CD8+ cytotoxic T cells is important in the antitumor immune response. The use of immunomodulators in antitumor treatment is increasingly common, with carboxymethyl-glucan (CM-G) being one of the best-described immunostimulators (192, 234). Magnani et al. (192) evaluated the CD3+, CD4+ and CD8+ cell populations of patients with advanced prostate cancer and compared this data with the *CCR5* genotype, associating it with the administration of oral CM-G for 28 days. The CCR5Δ32 variant was found only in a heterozygous genotype, in six patients, at an allelic frequency of 10%. Five patients reported a family history of prostate cancer, two of whom had affected first-degree relatives. Both patients carried the CCR5Δ32 allele. In general, CCR5Δ32 non-carriers had higher counts on CD3+ and CD4+ cells when comparing respectively after and before treatment with CM-G, as well as higher counts of CD8+ cells when comparing to CCR5Δ32 carriers only after treatment with CM-G. In addition, the average CD4+/CD8+ cell ratio showed a worsened antitumor response after treatment in CCR5Δ32 allele carriers (192). Zambra et al. (193) also evaluated the CCR5Δ32 frequency in Brazilian prostate cancer patients, comparing to individuals affected by benign prostatic hyperplasia and healthy subjects. No association was found considering the variant and risk to both conditions, nor with clinical outcomes (193).

Aoki et al. (188) assessed the CCR5Δ32 frequency in individuals with breast cancer and healthy women. However, no significant difference was observed between groups. The impact of *p53* genotypes, a known tumor suppressor gene, together with the CCR5Δ32 genotypes, was also evaluated revealing a higher frequency of individuals with the p53 Arg homozygous genotype and the CCR5Δ32 wild-type genotype amongst controls as compared to patients (188). Banin-Hirata et al. (189) also evaluated whether the CCR5Δ32 variant was associated with susceptibility, response to treatment, and clinical course of breast cancer. No association was found between CCR5Δ32 and the features analyzed (189). In accordance, Derossi et al. (190) did not found an association between the CCR5Δ32 and CCL5 levels in breast cancer (190).

HPV infection is the main cause of cervical cancer. However, factors other than HPV infection, including genetic, immune, and environmental factors, also affect tumorigenesis (159, 235, 236). In this context, Santos et al. (159) evaluated the CCR5Δ32 frequency in HPV+ women with and without cervical neoplastic lesions. No association was found between the variant and the presence of cancer or lesions severity (159).

In addition to the multiple roles of CCR5 in tumorigenesis and antitumor response, this molecule is also an important modulator of neuroinflammation (237–239), potentially affecting the development of brain-related diseases. In this sense, Vieira-Filho et al. (191) found an association between the presence of the CCR5Δ32 allele and susceptibility to neuroblastoma (191). Lastly, Oliveira et al. (187) investigated the role of the CCR5Δ32 variant in acute lymphoblastic leukemia, but no association was found between the variant and the disease development (187). In conclusion, the CCR5 has varied influences in different types of cancer.

## Impacts of CCR5Δ32 on a Highly Admixed Population – A Critical Look

At a population level, the effects of CCR5Δ32 on European populations may be different than those potentially observed in highly admixed populations. However, the population-specific effects of CCR5Δ32 are not only due to its frequency, but also due to its interaction with different alleles. There are nine widely known *CCR5* haplotypes, which are formed by combinations of eight *CCR5* polymorphisms (including CCR5Δ32) and one polymorphism located in the *CCR2* gene (40, 70). The impact of the *CCR5* haplotypes on HIV disease progression differs between African Americans and Caucasians since the effects of the CCR5Δ32 can be modulated by other alleles heterogeneously distributed among the populations (240). In a broader perspective, this information indicates that the effect of the CCR5Δ32 observed in Europeans (or other non-Brazilian populations) may be modified by further genetic traits circulating in Brazilians, which may also vary in different regions of the country. In fact, the detection of the real effect of CCR5Δ32 on different health and disease conditions in the Brazilian population is not a simple task. Of note, gene-disease association studies performed with admixed populations can be difficult due to differential linkage disequilibrium patterns (241).

Pharmacogenomic approaches, including the use of CCR5 modulators based on the CCR5Δ32 genotyping, must be considered at an individual level, especially in highly admixed populations, where the frequency of polymorphisms may be quite different from those observed in populations with greater genetic homogeneity (7). The CCR5Δ32 genotyping could be considered in pharmacological treatments involving CCR5 blockade in the context of inflammatory diseases or types of cancer. The use of CCR5 modulators in individuals with the CCR5Δ32 genotype probably has a limited effect due to the natural absence of CCR5 expression on the cell surface. Although the number of individuals with this genotype is exceptionally low in an admixed population such as the Brazilian population, the cost-benefit of this strategy must be considered on a case-by-case basis. Despite the limitations, the area of pharmacogenomics involving CCR5Δ32 genotyping is expected to progress in the next years, especially considering the increasing use of CCR5 modulators to treat other diseases not associated with HIV infection. Some important advances have already been made. For instance, the CCR5Δ32 genotyping can help clinicians to predict the progression of human enteroviral cardiomyopathy, also helping the decision making concerning the early use of antiviral interferon-β therapy in such condition (242).

## Conclusions

The CCR5Δ32 allele frequency is quite variable in Brazil, being extremely low in some regions (e.g., 0.6% in Rondônia), but high in others (e.g., up to 9.3% in Paraná and 7.4% in Rio Grande do Sul). In Native American populations, the allele is absent or occurs at low frequencies. In Brazil, CCR5Δ32 is not uncommon in non-Caucasian populations, because of the miscegenation that has occurred in the country.

Many studies corroborated the protective effect of the CCR5Δ32 on susceptibility or clinical aspects of HIV infection in the Brazilian population. On the other hand, there is no evidence pointing to a relevant role for CCR5Δ32 on Cutaneous leishmaniasis, Chagas disease, HTLV-1, Dengue virus, Influenza A, HPV, HBV and HCV infections, or HCV-HIV co-infection in Brazilians. Limited evidence indicates a potential involvement of CCR5Δ32 wild-type genotype in ocular toxoplasmosis and a protective effect of the variant on HBV/HIV co-infection.

Considering inflammatory conditions, the CCR5Δ32 can influence both the susceptibility and the clinical outcome of multiple sclerosis. Of note, CCR5Δ32 reduces the risk of preeclampsia and periodontitis development, potentially due to the CCR5Δ32-associated reduced inflammation. Moreover, CCR5Δ32 can reduce the risk of rheumatoid arthritis, but contributes to the progression and clinical status of juvenile idiopathic arthritis patients. CCR5Δ32 can also influence sickle cell anemia-related immune conditions. However, the impact of CCR5Δ32 on systemic lupus erythematosus is controversial. Concerning tumoral development, the CCR5Δ32 has varying influences on the development of different types of cancer, including prostate cancer and breast cancer. It is not possible to generalize the impact of the variant on cancer development, especially in the Brazilian population.

Understanding the real impact of the CCR5Δ32 variant in different conditions is essential to indicate in which diseases the use of CCR5 modulators may be relevant. This knowledge is fundamental for the advancement of CCR5-based therapies, especially in populations with a complex genetic structure. Finally, CCR5Δ32 influences should be assessed within the context of each population, since genetic admixture and interactions with other alleles may alter the expected phenotypic effects attributed to CCR5Δ32.

## Author Contributions

BK-L and JE wrote the first version of the manuscript. JC revised and edited the text. All authors contributed to the article and approved the submitted version.

## Funding

BK-L receives a fellowship from Conselho Nacional de Desenvolvimento Científico e Tecnológico (CNPq, Brazil). JE receives a postdoctoral fellowship from Coordenação de Aperfeiçoamento de Pessoal de Nível Superior (Programa Nacional de Pós-Doutorado – PNPD/CAPES, Brazil). JC receives a research fellowship from CNPq (Bolsa de Produtividade em Pesquisa - Nível 1A, Brazil) and has research projects funded by Fundação de Amparo à Pesquisa do Estado do Rio Grande do Sul (FAPERGS, Brazil) and CAPES (Brazil).

## Conflict of Interest

JE is Guest Associate Editor of Frontiers in Immunology but he was not involved in editing this article.

The remaining authors declare that the research was conducted in the absence of any commercial or financial relationships that could be construed as a potential conflict of interest.

## Publisher’s Note

All claims expressed in this article are solely those of the authors and do not necessarily represent those of their affiliated organizations, or those of the publisher, the editors and the reviewers. Any product that may be evaluated in this article, or claim that may be made by its manufacturer, is not guaranteed or endorsed by the publisher.
